# Vagal Oxytocin Receptors as Molecular Targets in Gut–Brain Signaling: Implications for Appetite, Satiety, Obesity, and Esophageal Motility—A Narrative Review

**DOI:** 10.3390/ijms26167812

**Published:** 2025-08-13

**Authors:** Agnieszka Nowacka, Maciej Śniegocki, Ewa A. Ziółkowska

**Affiliations:** 1 Department of Neurosurgery, Collegium Medicum in Bydgoszcz, Nicolas Copernicus University in Toruń, ul. Curie Skłodowskiej 9, 85-094 Bydgoszcz, Poland; a.nowacka@cm.umk.pl (A.N.); sniegocki@cm.umk.pl (M.Ś.); 2 Department of Pediatrics, School of Medicine, Washington University in St. Louis, St. Louis, MO 63110, USA

**Keywords:** oxytocin, oxytocin receptor, vagus nerve, gut–brain axis, gastrointestinal motility, metabolic regulation, GLP-1, chemogenetics, dysphagia, obesity

## Abstract

Oxytocin (OT), traditionally associated with reproduction and social bonding, has emerged as a key modulator of gastrointestinal (GI) physiology and appetite regulation behavior through its actions within the gut–brain axis. Central to this regulation are vagal oxytocin receptors (VORs), which are located along vagal afferent and efferent fibers and within brainstem nuclei such as the nucleus tractus solitarius and dorsal motor nucleus of the vagus. This review presents a comprehensive synthesis of current knowledge on the anatomical distribution, molecular signaling, developmental plasticity, and functional roles of VORs in the regulation of GI motility, satiety, and energy homeostasis. We highlight how VORs integrate hormonal, microbial, and stress-related cues and interact with other neuropeptidergic systems including GLP-1, CCK, and nesfatin-1. Recent advances in spatial transcriptomics, single-nucleus RNA sequencing, chemogenetics, and optogenetics are discussed as transformative tools for mapping and manipulating VOR-expressing circuits. Particular attention is given to sex differences, translational challenges, and the limited understanding of VOR function in humans. This article proposes VORs as promising therapeutic targets in dysphagia, obesity, and functional GI disorders. We outline future research priorities, emphasizing the need for integrative, cross-species approaches to clarify VOR signaling and guide the development of targeted, personalized interventions.

## 1. Introduction

Oxytocin, a neuropeptide traditionally recognized for its roles in parturition and social bonding, has garnered increasing attention for its diverse physiological functions extending well beyond reproductive and affiliative behaviors. Its widespread distribution throughout the central nervous system (CNS) and peripheral tissues underscores a multifaceted involvement in homeostatic regulation, including the modulation of gastrointestinal (GI) physiology, pain transmission, and appetite control [1,2,3,4]. The presence of oxytocin receptors (OTRs) in various regions of the gut and brain, as well as on vagal afferent neurons, highlights the complexity of oxytocinergic signaling within the gut–brain axis [5,6]. Recent research highlights the significance of oxytocinergic pathways in regulating GI motility, secretion, visceral sensitivity, and satiety signaling. OTR expression in the intestinal epithelium and vagal sensory neurons suggests roles in gut maturation, barrier integrity, and nutrient sensing [4,5,7]. Vagal afferents serve as key conduits, transmitting oxytocin-modulated peripheral signals to the nucleus tractus solitarius (NTS) and other brainstem nuclei, thereby influencing appetite and energy balance [3,8,9,10]. Furthermore, oxytocin interacts with regulatory peptides such as GLP-1, CCK, and nesfatin-1, positioning it as a central player in hormonal and neural integration along the gut–brain axis.

Despite these advances, the specific role of vagal oxytocin receptors (VORs), a subset of OTRs located on vagal afferents, efferents, and brainstem autonomic neurons, remains poorly understood. While several studies suggest that VORs modulate esophageal motility, satiation, gastric reflexes, and thermogenic responses, their precise anatomical distribution, cellular identity, downstream signaling, and translational relevance, particularly in humans, are still unclear [11,12,13,14]. Moreover, conventional oxytocin delivery strategies, such as intranasal administration, may not adequately reach these peripheral receptors, limiting therapeutic efficacy in GI disorders and highlighting the need for novel targeting approaches [4,6,15,16].

A clear distinction must be made between central and peripheral effects of oxytocin (OT). Central OT actions are mediated via oxytocin receptors in brain regions such as the paraventricular nucleus (PVN), amygdala, and nucleus tractus solitarius (NTS), influencing behavior, emotion, and autonomic output [2]. Peripheral OT exerts significant modulatory effects on gastrointestinal motility and autonomic functions through oxytocin receptors on vagal afferents, enteric neurons, and intestinal epithelial cells [4,5]. Vagal oxytocin signaling acts as a brake on intestinal peristalsis and reduces enteric neuronal activation, while oxytocin receptors in vagal nerves are essential for esophageal motility and function [6]. The hypothalamic–vagal oxytocinergic neurocircuitry plays a crucial role in modulating gastric emptying and motility, particularly following stress [7]. The extent to which intranasally administered OT accesses the central nervous system remains actively debated. While some studies suggest limited direct CNS penetration via nose-to-brain pathways, emerging evidence indicates that intranasal OT may influence brain function through multiple routes: direct CNS entry, peripheral receptor activation with subsequent vagal signaling to the brainstem, and systemic effects via increased plasma oxytocin levels. Notably, the peripheral administration of oxytocin can suppress addictive behaviors through vagal mechanisms, demonstrating the therapeutic potential of peripheral oxytocin signaling [3]. These findings have important implications for therapeutic strategies, including the development of oral oxytocin analogues for treating abdominal pain [16] and personalized psychiatric therapy approaches using intranasal oxytocin [15], suggesting that targeting peripheral vagal oxytocin signaling may be therapeutically viable even when direct CNS penetration is limited.

This narrative review addresses these critical knowledge gaps by providing the first comprehensive synthesis of VOR structure, function, and therapeutic potential within the gut–brain axis. We focus on several inter-related domains: developmental and region-specific expression of VORs; intracellular signaling cascades and second-messenger pathways; modulation by sex hormones, immune signals, and microbiota-derived metabolites; and roles in appetite regulation, esophageal and intestinal motility, and metabolic homeostasis. We also examine how cutting-edge technologies, such as spatial transcriptomics, single-nucleus RNA sequencing, chemogenetics, and advanced imaging, are transforming our understanding of VOR-expressing circuits. By integrating neurobiological, physiological, and translational perspectives, this review offers a novel framework for understanding vagal oxytocin receptors as therapeutic targets in disorders such as dysphagia, obesity, and functional GI syndromes.

## 2. Peripheral Oxytocin in Metabolic and Gastrointestinal Regulation

Oxytocin (OT) is a neuropeptide best known for its roles in childbirth and lactation. However, OT also acts far beyond reproduction, influencing gastrointestinal physiology, metabolism, and the gut–brain axis. Oxytocin receptors are expressed not only in the uterus and mammary glands, but also in peripheral tissues like the GI tract, heart, and vagus nerve, including both afferent and efferent fibers [4,6,17,18,19]. Through these pathways, OT helps regulate gut motility, satiety, energy balance, immune signaling, and even visceral pain. Functionally, OT reduces spontaneous colonic contractions, shortens gastrointestinal transit time, and supports coordinated intestinal movement [2,20,21]. These effects are partly mediated by vagal circuits. Vagal afferents detect circulating OT and signal to the brain, influencing food intake and energy regulation. The loss of vagal OTR signaling impairs esophageal transit and swallowing [6], highlighting its relevance for motility disorders. OT also affects metabolism through its action on hypothalamic and hindbrain satiety pathways. It suppresses appetite, reduces caloric and fat intake, and counters leptin resistance [22,23,24,25]. At the same time, OT boosts thermogenesis in brown adipose tissue (BAT) by upregulating UCP1, linking vagal signaling to energy expenditure [26,27]. Cold exposure enhances this effect by increasing OT and OTR expression in both central and peripheral tissues. The neuroimmune and sensory roles of OT are also becoming clearer. OT is expressed in pain-processing regions of the spinal cord and modulates visceral hypersensitivity, suggesting therapeutic potential for functional GI disorders. In the gut, OT interacts with the enteric nervous system, immune cells, and microbiota, forming a complex regulatory network. Microbial signals and other peptides like CGRP and VIP can shape OT activity, further tying it to the gut–brain–immune axis [28,29,30]. Despite promising effects, there are still challenges. OT has a short half-life, poor GI stability, and limited brain penetration. Some peripheral OTRs may lie beyond the reach of intranasal delivery, prompting efforts to develop analogs or targeted delivery systems [4,31]. OT’s uterotonic effects also raise concerns for its use in reproductive-age women [6]. Overall, OT integrates digestive, metabolic, and thermoregulatory functions through peripheral and vagal pathways. This makes it a compelling therapeutic target for obesity, dysphagia, and GI disorders, though more work is needed to refine delivery strategies and account for sex differences, tissue-specific actions, and complex neuroimmune interactions.

## 3. The Vagus Nerve as a Central Gut–Brain Interface for Oxytocin Signaling

The vagus nerve, or cranial nerve X, is a key pathway linking the digestive system with the brain. It plays a central role in bidirectional gut–brain communication, helping the brain monitor and adjust digestive activity in real time. About 80–90% of its fibers are sensory, sending continuous feedback from the esophagus, stomach, and intestines to brainstem regions such as the nucleus tractus solitarius (NTS) [4,6,7,10,32]. These signals reflect mechanical stretch, nutrient content, hormonal input, immune status, and even microbial metabolites, allowing the brain to regulate satiety, digestion, inflammation, and energy balance [10,33,34]. Oxytocin receptors (OTRs) are expressed on many of these vagal sensory neurons, especially within the nodose ganglia, enabling oxytocin to directly influence how peripheral signals are processed [7,26,35,36,37]. These same neurons also respond to gut peptides such as CCK, GLP-1, and nesfatin-1, and can integrate inflammatory or microbial cues [11,38,39,40,41]. Some vagal afferents form synapses directly with enteroendocrine cells in the gut lining, using glutamate to rapidly transmit nutrient-related information to the brain. Others sense microbial products like short-chain fatty acids that modulate neuronal excitability and influence host physiology [8,10,40,42,43,44]. The integration of these diverse inputs positions vagal sensory neurons as central hubs in neuroimmune and metabolic control. The vagus nerve also contains motor fibers that originate from the dorsal motor nucleus of the vagus (DMV) and innervate smooth muscles in the esophagus, stomach, and intestines. These efferent fibers coordinate motility and secretions essential for digestion [10,41,45]. Although most oxytocin receptors are found on sensory neurons, oxytocin released from hypothalamic nuclei can influence vagal motor output indirectly, for example, by enhancing parasympathetic tone or modifying synaptic inputs to DMV neurons [6,26,46,47]. This interplay is important for regulating reflexes such as swallowing and esophageal peristalsis. At the neurochemical level, vagal neurons express a rich array of receptors and signaling molecules that allow them to respond to internal and external stimuli. These include not only OTRs but also receptors for ghrelin, CCK, GLP-1, and other gut-derived peptides [5,48,49,50,51,52]. The activity of these neurons can be modulated by sex hormones, immune cytokines, and environmental cues such as cold exposure [24,46,53,54]. Functional studies have shown that manipulating vagal sensory neurons, particularly those expressing OTRs, alters food intake, gut motility, and energy expenditure [6,24,41,54,55]. Despite these insights, the specific identity and function of oxytocin-responsive vagal subpopulations remain poorly understood. Researchers have begun using advanced tools, such as single-cell RNA sequencing, genetic labeling, chemogenetics, and optogenetics, to map these circuits with greater precision and link molecular profiles to functional outputs [37,41,54]. These approaches are crucial for identifying which vagal neurons express OTRs and for understanding how vagal oxytocin signaling contributes to digestive health, mood regulation, and metabolic homeostasis.

## 4. Vagal Oxytocin Receptors: Localization, Specialization, and Plasticity

Vagal oxytocin receptors (OTRs) play a central role in translating peripheral oxytocin signals into brain-mediated responses that regulate digestion, appetite, and energy balance. These receptors are distributed across vagal sensory and motor pathways and influence how the nervous system integrates signals from the gastrointestinal (GI) tract. However, their distribution, function, and regulation are not uniform.

### 4.1. Distribution and Neuronal Diversity of Vagal Oxytocin Receptors

Oxytocin receptors (OTRs) are present on both sensory and motor fibers of the vagus nerve and play a key role in gut–brain communication. Their distribution is not uniform, and certain subtypes of vagal neurons, especially those in the nodose ganglia, express more OTRs, which may explain why oxytocin affects processes like swallowing, satiety, and gut motility [4,6,7,24,36,56]. These sensory neurons detect signals from the gut and transmit them to the brain. When oxytocin signaling is disrupted, especially in esophagus-associated fibers, food passage is impaired, an effect more pronounced in males [6]. Central vagal terminals in the brainstem, such as those projecting to the nucleus tractus solitarius (NTS), also express OTRs. Oxytocin applied here can influence gastric motility and stress-related gastric delay [7,20,24,30,57,58]. Although many vagal neurons express OTRs, we still lack a full map of their subtype-specific localization. Single-cell RNA sequencing and imaging tools are starting to fill this gap [30,59,60,61]. These neurons likely respond not only to oxytocin but also to other peptides, including cholecystokinin (CCK) and nesfatin-1, contributing to a complex regulatory network [37,43,62,63,64].

### 4.2. Regional and Functional Specialization

Vagal neurons differ depending on which part of the gastrointestinal tract they innervate. Some respond to gut stretch, others to hormones or nutrients, and their roles vary across regions like the esophagus, stomach, and colon [7,10,54,65]. OTRs are not evenly distributed, neurons involved in swallowing and esophageal control often lie outside the brain, limiting the effect of intranasal oxytocin [4,6,41]. Moreover, the same dose of oxytocin may have different effects in the proximal versus distal colon, likely due to local differences in receptor types and ion channel expression [3,20,63]. Interactions between gut-derived signals like CCK, microbial metabolites, and gut stretch create region-specific vagal responses [48,49,66]. The recycling of OTRs after activation, internalization and re-expression on the membrane, also varies and may influence oxytocin sensitivity over time [4,7,24,56]. This regional diversity and receptor dynamics are important when designing targeted therapies.

### 4.3. Developmental and Contextual Plasticity of Vagal Oxytocin Signaling

The expression and function of OTRs in vagal neurons exhibit remarkable plasticity, being dynamically regulated by developmental stage, hormonal milieu, environmental factors, and pathological conditions. OTRs appear early in both enteric and vagal systems during embryonic development, helping establish gut–brain circuits during critical windows that influence lifelong responsiveness [5,41,55,65,67,68]. Early life experiences, including maternal care, feeding patterns, and environmental stressors, permanently alter OTR expression and sensitivity in vagal circuits, creating individual differences in therapeutic responsiveness that persist into adulthood [37,43]. Sex hormones profoundly modulate this system, with estrogen upregulating colonic OTR expression and creating cyclical variations in sensitivity throughout the menstrual cycle, potentially explaining sex differences in gastrointestinal disorders and oxytocin therapy responses [66,69,70]. Environmental exposures during development, particularly high-fat diets, fundamentally reprogram vagal neuron responsiveness to oxytocin, leading to impaired gastric signaling and disrupted satiety mechanisms [24,34,48,65,71,72]. Chronic stress, metabolic dysfunction, and inflammatory conditions further modify vagal oxytocin sensitivity through complex interactions involving immune signaling, gut barrier function, and microbiota-derived metabolites [1,7,10,19,30,40,48].

This plasticity presents both opportunities and challenges for therapeutic development, suggesting that oxytocin-based interventions must be tailored to individual patient characteristics including age, sex, developmental history, metabolic status, and immune state [15,31]. Disease states such as gastroparesis, functional dyspepsia, sleep disorders, and aging create distinct patterns of altered vagal oxytocin signaling that may require specialized therapeutic approaches [7,12,14,56]. The developmental programming of these systems indicates that early interventions might be particularly effective, while the persistence of developmental influences means adult treatments must account for established patterns of oxytocin responsiveness [16,31,73]. Future therapeutic strategies should focus on identifying predictive biomarkers, developing targeted delivery systems for specific gut regions or vagal populations, and designing combination therapies that address multiple aspects of altered physiological states. Understanding this plasticity will be key to designing therapies that align with the patient’s age, sex, gut region, and immune or metabolic status, ultimately enabling personalized gastrointestinal medicine based on individual patterns of vagal oxytocin system function.

### 4.4. Functional Mapping and Connectivity

#### 4.4.1. Integration with Brainstem Nuclei

Building on their distribution along vagal fibers, oxytocin receptors (VORs) contribute to central autonomic control by integrating with brainstem nuclei involved in digestion and appetite regulation. Hypothalamic paraventricular nucleus (PVN) neurons project into the dorsal motor nucleus of the vagus (DMV) and nucleus tractus solitarius (NTS), forming a critical circuit through which oxytocin modulates vagal output and upper GI reflexes [26,37,47,56,58,74]. Within this loop, VORs help fine-tune responses to physiological challenges, including those related to swallowing, satiety, and gastric function. For instance, nesfatin-1 interacts with oxytocin signaling to modulate the swallowing reflex in rats, suggesting functional interplay between these systems in brainstem circuits [75,76,77,78]. Oxytocin also acts centrally to influence appetite regulation. In animal models, central oxytocin administration reduces food intake independently of classical receptor blockade, pointing to complex, layered regulatory mechanisms [26,35,36,37,79,80]. In humans, oxytocin enhances neural activity linked to reward and self-regulation, likely initiated at the level of brainstem vagal relay centers [80]. Disruptions in this brainstem–vagal interface are associated with impaired gastric emptying and swallowing, underscoring the translational potential of targeting VOR circuits in clinical settings [6,10,37,41]. Importantly, oxytocin’s effects vary depending on the site of action, central versus peripheral, highlighting the precision required in therapeutic modulation [3,34,46,81]. However, the exact brainstem neuronal populations expressing VORs and their connectivity remain incompletely defined, warranting further investigation using optogenetics, circuit tracing, and single-cell profiling tools.

#### 4.4.2. Peripheral Connectivity to Gastrointestinal Organs

**Figure 1:**
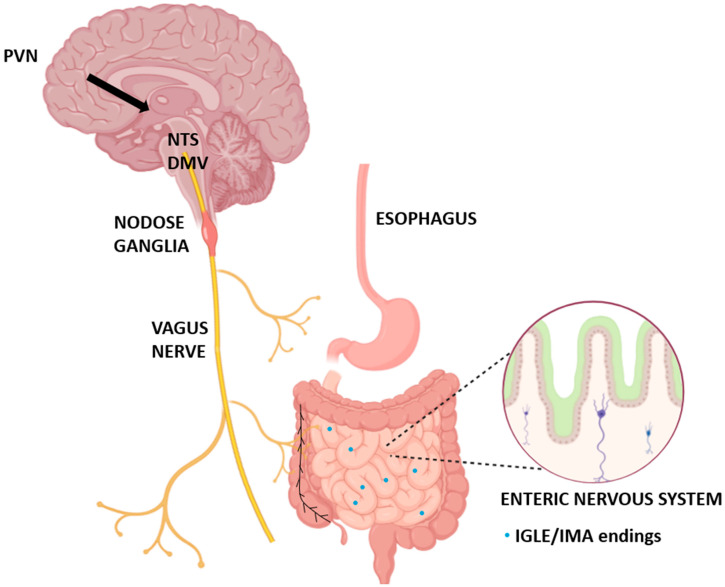
Anatomical localization of vagal oxytocin receptors (VORs). Schematic illustration showing central and peripheral components of the vagus nerve involved in oxytocin signaling. Projections from the paraventricular nucleus (PVN) of the hypothalamus innervate brainstem nuclei, including the dorsal motor nucleus of the vagus (DMV) and nucleus tractus solitarius (NTS). Vagal afferent fibers project from the gastrointestinal tract into nodose ganglia and the brainstem, while efferent fibers reach the esophagus and intestines. The enteric nervous system (ENS) contains intraganglionic laminar endings (IGLEs) and intramuscular arrays (IMAs), which serve as peripheral targets of vagal sensory fibers expressing oxytocin receptors.

In the periphery, VORs are positioned along vagal afferents and efferents innervating the esophagus, stomach, intestines, and colon, allowing for region-specific modulation of motility and visceral sensitivity [6,28,73,82]. PVN projections to the DMV influence parasympathetic outflow, including cardiac vagal neurons, and release oxytocin in a context-dependent manner [38,39,83,84]. Beyond neurons, OTRs are also expressed in gut epithelial and smooth muscle cells. Welch et al. [5] demonstrated OTR immunoreactivity in villus and crypt epithelium, while Wang et al. [20] showed that oxytocin locally inhibits contractions in both the duodenum and colon, suggesting direct effects on gastrointestinal muscle tone. This peripheral vagal network exhibits anatomical and functional specialization. Different vagal neuron subtypes project into discrete gut regions, for example, GLP1R-expressing afferents preferentially target the stomach, while others reach intestinal villi [47,85,86,87,88]. This anatomical divergence likely reflects functional heterogeneity within the oxytocin–vagal axis. Vagal signaling is further shaped by interactions with other neuropeptides, including CCK, POMC, and NPY, and can be modulated by emotional or endocrine states [8,30,52,58]. Additionally, peripheral OTRs may serve as sites for afferent feedback, relaying information from the gut to the brain [63]. Segmental differences also influence oxytocin’s effects. Rochman et al. [28] reported variable sensory neuron density across the esophagus, which may underlie region- and species-specific responses. Despite accumulating evidence, some inconsistencies remain. For example, Bülbül et al. [89] found no significant changes in gastric motility following either central or peripheral oxytocin application, indicating that context and experimental conditions critically shape outcomes. These findings underscore the need for precise mapping of VOR function across physiological and disease states using advanced, spatially resolved techniques. To provide a visual overview of the anatomical sites involved in vagal oxytocin signaling, including both central nuclei and peripheral targets, Figure 1 summarizes the key components of the gut–brain axis implicated in this pathway.

## 5. Intracellular Signaling and Neurochemical Interactions of Vagal Oxytocin Receptors

### 5.1. Receptor Pharmacology and Kinetics

Vagal oxytocin receptors, like other G protein-coupled receptors, respond to oxytocin through ligand binding and intracellular signaling that depends on the tissue context. OTR mRNA and protein have been detected in tissues innervated by the vagus nerve, suggesting local autocrine and paracrine regulation, though this requires confirmation by co-localization studies such as in situ hybridization or immunohistochemistry [39,73,82,90]. VOR expression and sensitivity depend on receptor density, local oxytocin concentration, and the presence of competing ligands or neurotransmitters. In the gastrointestinal tract, oxytocin influences motility independently of VIP, ATP, or adenosine pathways, as blocking these signals does not alter the oxytocin response [20,30,58,64,91]. This supports a distinct pharmacological profile for VORs in gut tissues. Activation of VOR typically triggers Gq/11-coupled pathways involving phospholipase C and calcium mobilization, which may lead to contraction or relaxation of smooth muscle depending on the cellular environment. Similar mechanisms are likely active in gut muscle layers, as in other oxytocin-sensitive tissues. Circulating oxytocin may also influence vagal output indirectly through central action, crossing the blood–brain barrier and modulating hypothalamic circuits such as those in the paraventricular nucleus [30,36,59,71,92]. In the PVN, oxytocin may modulate neuroendocrine activity, including corticotropin-releasing factor (CRF) secretion, although it remains unclear whether OTRs are directly expressed on CRF-producing neurons [48]. Within the nodose ganglia, the spatial organization of vagal afferents contributes to the specificity of oxytocin signaling [88]. From a translational perspective, oxytocin can affect appetite, thermogenesis, and body weight. However, whether these effects are primarily mediated centrally or peripherally is still debated [27,67]. Activation of Oxtr^+^ neurons appears to suppress feeding behavior without broadly affecting other actions, which makes these pathways attractive for therapeutic targeting [54]. Importantly, receptor-level mechanisms such as desensitization, internalization, and recycling may explain functional changes in motility more than plasma oxytocin levels alone. For example, altered VOR responsiveness may contribute to disorders like slow transit constipation [71].

### 5.2. Downstream Signaling Pathways

#### 5.2.1. Canonical GPCR Signaling

**Figure 2:**
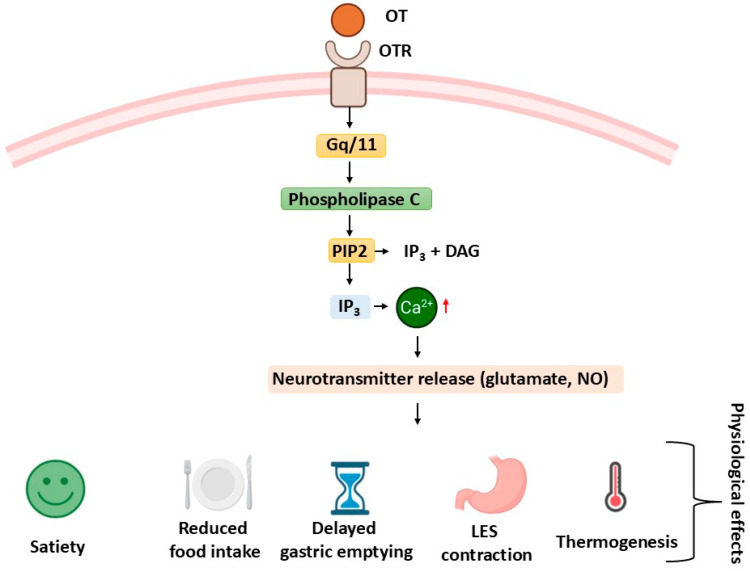
Canonical Gq/11–PLC–Ca^2+^ signaling pathway of vagal oxytocin receptors and their physiological effects. Upon oxytocin (OT) binding, the oxytocin receptor (OTR) activates Gq/11 proteins, which stimulate phospholipase C (PLC). PLC hydrolyzes PIP_2_ into IP_3_ and DAG, leading to intracellular Ca^2+^ release. Elevated calcium levels promote the release of neurotransmitters such as glutamate and nitric oxide (NO), resulting in diverse physiological outcomes. These include satiety, reduced food intake, delayed gastric emptying, lower esophageal sphincter (LES) contraction, and thermogenesis. This figure summarizes the canonical signaling cascade and downstream effects mediated by vagal OTR activation.

Vagal oxytocin receptors signal primarily through G protein-coupled receptor (GPCR) pathways, with oxytocin binding triggering a conformational change in the receptor. Most OTRs activate Gq/11 proteins, leading to phospholipase C (PLC) stimulation, cleavage of PIP2, and generation of IP3 and DAG. These second messengers release intracellular calcium and activate protein kinase C (PKC), which influences vagal neuron excitability and gene expression [14,82,93,94,95]. This signaling cascade is a key mechanism by which oxytocin modulates afferent input from the gut to the brain. In addition to Gq/11, OTRs can couple to Gi or Gs proteins, allowing for diverse downstream effects on cAMP signaling depending on cell type and context [14,64]. This signaling flexibility helps explain why VOR responses differ across gastrointestinal regions. Structural data suggest that oxytocin stabilizes the receptor’s active conformation, while antagonists such as retosiban block this process [4]. Small structural distinctions from vasopressin receptors enable signaling specificity and reduce off-target effects [68]. Other regulatory features include receptor internalization, heterodimerization with other GPCRs, and interactions with scaffolding proteins that modulate signal duration and intensity. Importantly, OTRs are co-expressed with other receptor families in vagal sensory neurons, forming part of a dense chemosensory network responsive to various gut-derived inputs [88]. Understanding these GPCR-based mechanisms provides a foundation for targeting VORs in disorders like dysphagia and obesity [4,96]. The canonical signaling pathway initiated by VOR activation is illustrated in Figure 2, highlighting the Gq/11–PLC–IP_3_–Ca^2+^ cascade and its downstream effects on neurotransmitter release and gastrointestinal physiology.

#### 5.2.2. MAPK and NO Signaling: Alternative Intracellular Pathways

Beyond classic G protein coupling, VORs may also activate intracellular cascades such as the mitogen-activated protein kinase (MAPK) and nitric oxide (NO) pathways. Although direct VOR involvement is still under investigation, MAPK activation by oxytocin is well documented and regulates neuronal plasticity, transcriptional programs, and long-term appetite control [22,67]. In obesity models, for example, central oxytocin reduces food intake even in the presence of leptin resistance, potentially via hindbrain MAPK signaling. The NO pathway, while less explored in the context of VORs, is vital for smooth muscle relaxation and gastrointestinal motility. Nitric oxide, as a major enteric neurotransmitter, mediates esophageal peristalsis and lowers sphincter tone [34,97]. Although direct evidence for VOR-triggered NO release is lacking, the anatomical proximity of vagal afferents to NO-producing cells supports potential indirect regulation [47,88,98,99]. Together, MAPK and NO systems may complement GPCR mechanisms, contributing to the context-specific modulation of motility and satiety. Their interplay with cholinergic and peptidergic inputs likely shapes integrated responses. Further work using transcriptomics and functional mapping is needed to define how these alternative pathways operate within VOR-expressing neurons [10,25,44,85].

#### 5.2.3. Network-Level Crosstalk with Neuromodulators

VORs operate within a broader neurochemical landscape shaped by multiple hormones and neurotransmitters involved in appetite, digestion, and reward. Among these, cholecystokinin (CCK) plays a central role by acting on vagal afferents to suppress food intake. Oxytocin and CCK both converge on downstream targets such as ERK1/2 in the hypothalamus, but may act via distinct initial pathways, highlighting parallel but independent mechanisms [24,100,101,102]. Other gut-derived hormones such as ghrelin, GLP-1, peptide YY, and neuropeptide Y signal through vagal sensory neurons that may also express OTRs. These neurons act as integrators of nutrient status and hormonal input. For instance, ghrelin promotes hunger and may interact with oxytocin signaling to influence appetite-related responses [8,103,104,105,106]. Oxytocin can also alter circulating ghrelin levels, though this depends on the delivery route and hormonal form [23]. Beyond the gut, VORs intersect with dopamine signaling in brain regions related to reward and motivation, influencing both homeostatic and hedonic eating [107]. GABAergic pathways also interact with oxytocin circuits; for example, oxytocin receptor-expressing nodose neurons may enhance activity in inhibitory GABA neurons in the area postrema and NTS. VORs thus function at the intersection of hormonal, mechanical, and sensory inputs. They help modulate not only digestion but also the emotional and motivational aspects of appetite and food intake. The co-expression of OTRs with receptors for CCK, GLP-1, dopamine, and GABA, combined with shared downstream signals such as ERK1/2, emphasizes the complex crosstalk that governs gut–brain communication [24,30,47,73,105,108,109,110].

#### 5.2.4. ADP-Ribosyl Cyclases and cADPR Signaling in Vagal and Intestinal Function

Beyond the canonical GPCR signaling cascades, oxytocin receptors may also interface with ADP-ribosyl cyclases and the cyclic ADP-ribose (cADPR) pathway, a less explored but functionally significant route for calcium mobilization. cADPR is a potent intracellular second messenger generated by the enzymatic activity of CD38 and CD157 (also known as BST1), which are widely expressed in the brain, intestine, and immune tissues. These ectoenzymes catalyze the conversion of NAD^+^ to cADPR, thus enabling calcium release from intracellular stores via ryanodine receptors, independently of classical PLC/IP_3_ signaling. Recent findings by Yahagi et al. [111] provide important insight into the physiological roles of this pathway. In CD38/BST1 double-knockout (DKO) mice, the authors reported a significant elongation of the small intestine, altered mesenteric lymph node cellularity, and disrupted immune homeostasis. Notably, CD38-deficient mice also displayed enhanced pyroptotic signaling via the TLR4–NLRP3–GSDMD axis, increased sensitivity to bacterial inflammation, and signs of autoimmunity, including elevated anti-dsDNA antibody titers and renal pathology. These effects are attributable to the loss of cADPR-mediated signaling and underscore CD38′s dominant enzymatic role at physiological pH. The cADPR system appears to contribute to both neural and immune regulation in the gut, and its crosstalk with oxytocin pathways may influence vagal excitability, B cell IL-10 production, intestinal barrier integrity, and motility. As such, the integration of oxytocin and cADPR signaling may represent a broader regulatory axis involved in digestive homeostasis and inflammation. Further research is warranted to determine whether vagal oxytocin receptors modulate or are modulated by cADPR pathways in a tissue-specific manner. Including this axis expands our understanding of vagus-linked oxytocin signaling beyond classical GPCRs, incorporating metabolic and immune-related feedback loops relevant to both physiology and disease.

### 5.3. Interactions with Gut Peptides and Hormones

#### 5.3.1. Integration of Vagal Oxytocin Receptors with Gut-Derived Peptides

**Figure 3:**
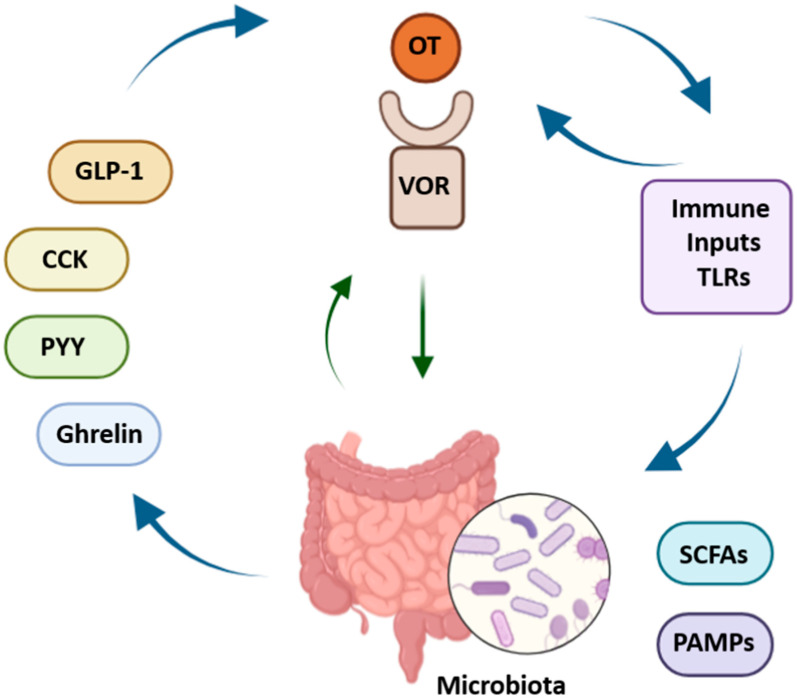
Integration of vagal oxytocin receptor (VOR) signaling with gut-derived peptides, immune inputs, and the microbiota. This schematic illustrates the bidirectional interactions between vagal oxytocin receptors (VORs), gut–brain peptides, microbial metabolites, and immune inputs. Oxytocin (OT) activates VORs, which modulate vagal afferent activity. VOR-expressing neurons are sensitive to key gut-derived hormones, such as GLP-1 (from the small intestine), CCK (duodenum), PYY (colon), and ghrelin (stomach), that regulate satiety, motility, and feeding behavior. Gut microbiota influence this signaling axis through short-chain fatty acids (SCFAs) and pathogen-associated molecular patterns (PAMPs), while Toll-like receptors (TLRs) on vagal afferents mediate the immune–microbial crosstalk. VOR signaling may also provide feedback to alter microbial composition or gut-derived peptide release, forming a dynamic gut–brain–microbiota circuit that governs the homeostatic control of digestion and metabolism.

Vagal oxytocin receptors (VORs) operate within a rich neurochemical environment shaped by gut-derived peptides such as glucagon-like peptide-1 (GLP-1), cholecystokinin (CCK), and ghrelin. These signals play key roles in regulating gastrointestinal motility, satiety, and energy homeostasis. GLP-1 is secreted postprandially and activates GLP1R-expressing vagal neurons that innervate the stomach and intestinal villi, facilitating the detection of stretch and nutrient presence [6,8,88,112,113,114,115]. This signaling cascade may modulate oxytocin responsiveness in both the nodose ganglion and the nucleus tractus solitarius (NTS), a key brainstem hub where inputs from GLP-1, CCK, oxytocin, and other peptides converge to shape digestive reflexes and satiety [52]. CCK is released during digestion and acts on vagal afferents through brainstem and hypothalamic circuits to inhibit appetite. The loss of CCK signaling impairs satiety, indicating its specific and non-redundant role. Vagal neurons that express oxytocin receptors appear responsive to both GLP-1 and CCK, suggesting the cooperative modulation of neuronal excitability and GI motor control [4,53]. Oxytocin itself reduces food intake and slows GI transit, likely through these vagal pathways [4]. Ghrelin, secreted by the stomach in response to fasting, stimulates appetite via vagal afferents that co-express ghrelin and oxytocin receptors—providing a mechanism to integrate hunger and satiety signals [10,106]. Interestingly, oxytocin’s anorexigenic effects seem largely independent of leptin and peptide YY (PYY), pointing toward distinct vagal or central mechanisms [23]. Vagal afferents transmit peptide signals to the brainstem primarily through glutamate. VOR activation may influence how signals such as ghrelin and CCK are processed in the dorsal vagal complex [10,97]. Furthermore, VOR-expressing neurons also respond to PYY and microbial signals via Toll-like receptors (TLRs), highlighting the convergence of metabolic, immune, and microbial cues in shaping vagal activity [10]. Together, these interactions illustrate the central role of VORs in integrating diverse peptide signals that regulate appetite, GI motility, and energy balance. These complex, bidirectional interactions between gut-derived peptides, vagal oxytocin receptors, immune signals, and the microbiota are summarized in Figure 3.

#### 5.3.2. Modulation of Vagal Neurotransmission by Oxytocin

**Figure 4:**
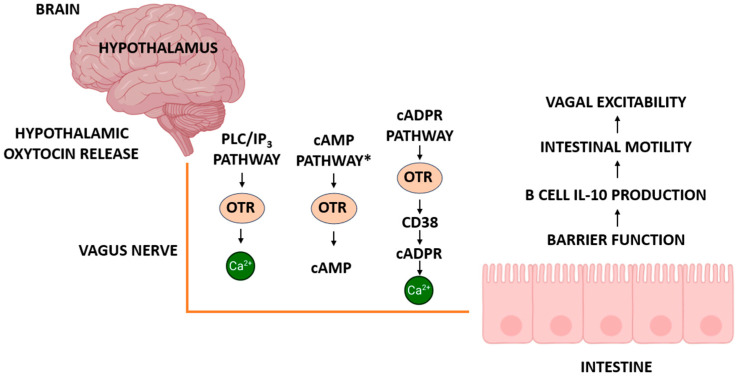
Oxytocin-related signaling pathways along the gut–brain axis. Hypothalamic oxytocin release activates vagal oxytocin receptors (OTRs), initiating multiple intracellular pathways that mediate gut–brain communication. These include the PLC/IP_3_–Ca^2+^ pathway, a context-dependent cAMP pathway, and the CD38–cADPR–Ca^2+^ axis. Downstream effects in the gastrointestinal tract include enhanced barrier function, increased IL-10 production by B cells, improved intestinal motility, and altered vagal excitability. These mechanisms contribute to the homeostatic regulation of digestion, inflammation, and appetite. (*) The cAMP pathway is context-dependent and may vary based on cell type, receptor conformation, and signaling environment.

VORs also regulate how vagal afferents transmit information from the GI tract to the brain, influencing excitatory and inhibitory neurotransmission. These receptors are expressed not only in vagal sensory neurons but also in gut tissues, including enteric neurons derived from the neural crest [5], supporting a direct role for oxytocin in afferent signal transmission. Vagal sensory neurons co-express receptors for GLP-1, CCK, and PYY peptides that are central to the modulation of appetite and GI function [30,54,59,106,116]. These hormones typically act through intracellular cascades that trigger the release of glutamate, the main neurotransmitter at the vagus–NTS synapse [10]. Oxytocin can fine-tune this excitatory transmission by modulating calcium signaling, receptor trafficking, and second-messenger pathways. It has also been shown to enhance CCK-driven responses, amplifying satiety signaling and vagally mediated motor reflexes [19,72,80]. In models of high-fat diet exposure, VOR activation in the nodose ganglion has been linked to altered satiety and stress pathways, underlining their role in metabolic adaptation [73]. Beyond excitatory control, oxytocin also modulates inhibitory neurotransmission. In the distal colon, it induces smooth muscle relaxation through nitric oxide (NO) release from nNOS-expressing neurons, engaging non-adrenergic, non-cholinergic (NANC) inhibitory pathways [19,20,57,117,118]. Through this dual regulation of excitatory and inhibitory signaling, VORs enable the precise control of GI motility and interoceptive feedback. By coordinating hormonal, neural, and immune signals at the synaptic level, these receptors support dynamic homeostatic adjustments essential for digestion, appetite, and energy regulation [5,46,72,73,106]. To provide a comprehensive overview of the mechanisms discussed in Section 5.2 and Section 5.3, we have included a schematic illustration summarizing the principal oxytocin-related signaling pathways along the gut–brain axis (Figure 4). This diagram integrates central and peripheral oxytocin release, major intracellular signaling cascades (PLC/IP_3_–Ca^2+^, context-dependent cAMP, and CD38–cADPR–Ca^2+^), and their downstream effects on gastrointestinal function and immune modulation.

## 6. Role of Vagal Oxytocin Receptors in Esophageal Motility

### 6.1. Esophageal Peristalsis and Swallowing

Esophageal peristalsis and swallowing depend on precise coordination between neural and muscular systems that ensure efficient transport of food from the mouth to the stomach. The vagus nerve plays a central role by integrating sensory input and driving motor responses, with growing evidence implicating vagal oxytocin receptors as key modulators of this process [9,39,88,119]. The esophagus hosts a dense network of immune cells, fibroblasts, antimicrobial peptides, Toll-like receptors (TLRs), and local microbiota, all interacting with neural and epithelial elements to maintain function [28]. VORs may contribute to this homeostasis by shaping vagal control of motility. Rodent studies show that disruption of oxytocin receptor signaling in the nodose ganglion results in food retention and impaired lower esophageal sphincter (LES) function, while ex vivo findings demonstrate that oxytocin promotes LES contraction, supporting a direct role in coordinating peristalsis [6]. Compared to neuropeptides like AVP or urocortin 3, oxytocin exerts especially strong effects on esophageal motility. Peripheral oxytocin enhances vagal afferent activity and esophageal contractility, improving swallowing efficiency [36,53]. These effects may be particularly beneficial in conditions such as obesity or diabetes, where oxytocin appears to restore vagal tone and esophageal performance. The exact distribution of VORs in esophageal innervation is still under investigation. The vagus nerve contains afferent and efferent fibers forming a feedback loop that integrates with other modulatory systems, including cholecystokinin and CART peptides [88]. Oxytocin may help compensate for regulatory deficits in disease states [53]. Neuronal heterogeneity further complicates this system, subsets of vagal afferents marked by vasoactive intestinal peptide (VIP) may have specialized roles in esophageal control [54], and clarifying their interaction with VORs will require loss-of-function and cell-specific studies. Recognizing VORs as central players in esophageal regulation opens new therapeutic possibilities for motility disorders. Oxytocin-sensitive interoceptive circuits within the nodose ganglion (NGOxtr) may also modulate esophageal function in stress-related or metabolic diseases [73]. Moreover, the interplay between immune signaling and neural control, as described by Rochman et al. [28], highlights further translational opportunities in immune-mediated esophageal dysfunction.

### 6.2. Regulation of Lower Esophageal Sphincter Function

The function of the lower esophageal sphincter (LES) depends on tightly coordinated sensory and motor signaling through the vagus nerve, particularly via the dorsal vagal complex. This brainstem region supports reflexive LES relaxation during swallowing and protects against gastroesophageal reflux. Disruptions in vagal signaling can impair LES relaxation or cause spastic contractions, contributing to motility disorders [6,120,121,122,123,124]. Recent insights highlight substantial heterogeneity among vagal sensory and motor fibers innervating the gut. Even neurons targeting the same organ can display divergent physiological properties. While traditional methods like electrical stimulation or pharmacological manipulation offer limited resolution, newer tools such as optogenetics and selective ablation now enable precise control over vagal subpopulations. For example, Chang [106] describes how closely intermingled vagal neurons can exert opposite visceral effects, supporting the idea of anatomically distinct labeled lines. This neuronal diversity is essential to understanding how oxytocin receptor (OTR)-expressing subsets contribute to LES regulation. Oxytocin signaling has been linked to gut motility, barrier function, and pain perception, offering therapeutic potential in conditions like irritable bowel syndrome and inflammatory bowel disease [4,19,57]. The presence of OTRs on vagal afferents and in the nodose ganglion provides a direct anatomical basis for oxytocin’s influence on LES control. Dantzler and Kline [56] report that OTR expression in the nodose ganglion increases under certain physiological challenges, although the specific roles and projections of these neurons remain unclear. Gut-derived signals add further complexity. The vagus nerve relays microbial and metabolic cues to the brain, which may in turn influence oxytocin-dependent regulation of the LES [10]. Although the exact mechanisms remain unclear, this likely involves interactions between vagal afferents, the nucleus tractus solitarius, and higher-order autonomic centers. For example, Everett et al. [3] show that failure of vagal afferents to respond to cholecystokinin (CCK-8) disrupts satiety signaling and LES function, underlining the importance of intact vagal circuits in satiety and esophageal reflex control. Targeting vagal oxytocin receptors could thus represent a promising therapeutic strategy for restoring normal LES activity in disorders such as dysphagia and obesity [6,56,106].

### 6.3. Sex-Specific Effects and Pathophysiology

Sex-specific differences in vagal oxytocin receptor (VOR) signaling and esophageal motility are increasingly studied, given the known sexual dimorphism of oxytocin pathways in both central and peripheral systems. Dumais and Veenema [69] report sex-specific patterns in oxytocin and vasopressin receptor distribution across species, suggesting distinct physiological roles in appetite regulation and autonomic function. Functional studies have yielded mixed findings. Asker et al. [6] showed that oxytocin administration reduces gastric emptying, but genetic ablation of oxytocin receptors (OTRs) in the nodose ganglion did not affect gastric transit in either sex. In contrast, esophageal motility appeared more sensitive: OTR knockdown impaired food transit equally in males and females, with no sex-dependent differences in severity. Similarly, ex vivo studies confirmed oxytocin-enhanced lower esophageal sphincter (LES) contraction without evidence of sex-specific effects. Nonetheless, sex hormones likely influence oxytocin signaling. Estrogens and androgens modulate OTR expression and function, potentially shaping downstream responses. For instance, Burmester et al. [25] found increased sucrose intake in oxytocin-deficient mice, an effect possibly mediated by gonadal steroids. Although direct links between sex hormones and vagal regulation of esophageal motility remain sparse, the possibility of modulation exists. Anatomical studies by Scott et al. [73] identified OTR-expressing neurons in the nodose ganglia projecting into the gut, suggesting a structural basis for sex-specific regulation. Liu et al. [4] also highlight that early-life microbiota and oxytocin signaling may diverge by sex, affecting gut–brain development. While current evidence shows no robust sex-dependent effects of VORs on esophageal function, study limitations may mask subtle differences. Investigating these potential distinctions remains crucial for advancing personalized medicine and developing sex-informed therapies for disorders like dysphagia and obesity.

## 7. Vagal Oxytocin Receptors in Appetite and Gastrointestinal Control

### 7.1. Peripheral Oxytocin and Satiety Signaling

Peripheral oxytocin (OT) plays a crucial role in satiety regulation through both its direct actions in the gastrointestinal (GI) tract and its modulation of vagal afferent signaling. OT and its receptors are widely distributed throughout the GI tract, excluding the caecum and gallbladder, supporting their broad involvement in digestive regulation [90]. In animal models, peripheral OT administration decreases food intake and body weight in a dose-dependent, receptor-mediated manner, as evidenced by the reversal of these effects with OT receptor antagonists [125,126]. The vagus nerve is essential in conveying gut-derived signals to the brain, facilitating OT’s satiety-enhancing effects [43]. Projections from the paraventricular nucleus (PVN) of the hypothalamus to brainstem regions, such as the nucleus tractus solitarius (NTS) and dorsal motor nucleus of the vagus (DMV), modulate vagal tone and influence appetite regulation [55]. Reduced expression of OT receptors (OTRs) in the NTS alters appetite regulation independent of vasopressin signaling [24]. Gut mechanical and chemical cues also stimulate vagal input, contributing to satiety via OT release [52]. OT interacts with various satiety hormones and may attenuate excitatory vagal signaling, reinforcing the sensation of fullness. In models of obesity, reduced serum OT and OTR expression correlate with increased fat mass and weight gain [27]. Intranasal OT has shown therapeutic potential by reducing food intake without central adverse effects [72]. These findings highlight peripheral OT as a promising target for modulating satiety and restoring energy balance [24,27,43,55,90,125,126].

### 7.2. Meal Size, Macronutrient Selection, and Patterns of Food Intake

Appetite regulation is shaped by intricate interactions between gut-derived signals and central neural circuits. Vagal oxytocin receptors are key mediators in this system, influencing not only meal initiation and termination but also preferences for specific macronutrients and eating schedules. Disruption of vagal signaling alters meal size and structure [43], while vagal afferents sense nutrient-specific and hormonal cues, including GLP-1, insulin, and nesfatin-1, many of which act locally due to limited blood–brain barrier permeability [36]. Recent studies reveal functionally distinct vagal subtypes involved in stretch detection and nutrient sensing, where oxytocin signaling may regulate meal size [54]. In both rodent and clinical contexts, OT administration consistently reduces food intake and promotes weight loss, including in Prader–Willi syndrome [36,67]. Chronic treatment enhances energy expenditure and metabolic efficiency, potentially independent of intake suppression [27,72]. Though its influence on macronutrient preference remains underexplored, VORs co-expressing neuropeptides like CART and responding to leptin and CCK may shape nutrient-driven choices [53]. Meal frequency and timing are also influenced by vagal afferents. Krieger et al. [51,127] demonstrated that these inputs affect satiation timing and inter-meal intervals, and their disruption may lead to erratic eating patterns. Intranasal OT improves metabolic markers in obese individuals, suggesting that VOR-mediated signaling also impacts meal regulation in humans [128].

### 7.3. Thermogenesis and Metabolic Regulation

Vagal oxytocin receptors (VORs) are emerging as critical modulators of thermogenesis and metabolic regulation. The vagus nerve integrates input from the gut and peripheral tissues, enabling hormones like GLP-1 and nesfatin-1 to influence central energy pathways [36,43]. Oxytocin activates vagal afferents at physiological concentrations, such as those present after feeding or during lactation, suggesting a role in coupling nutritional status to thermogenic output [53]. Beyond suppressing intake, OT promotes energy expenditure and fat oxidation, even in diet-induced obesity models [22,67]. These effects coincide with improved glucose and lipid homeostasis following peripheral OT delivery [27]. OTR expression in the myenteric plexus and colonic tissues supports peripheral involvement in these metabolic shifts [66]. At the vagal level, coordination with peptides like nesfatin-1 may modulate thermogenesis dynamically based on energy status [36]. However, the specific neuronal populations and downstream circuits involved remain to be fully mapped. Given its unique receptor pharmacology, OT presents a valuable therapeutic target for obesity and metabolic disorders [63].

### 7.4. Sex Differences and Behavioral Outcomes

Sex-specific aspects of the oxytocin (OT) system significantly influence behavioral and physiological responses. Differences in OT receptor distribution, downstream signaling, and receptor sensitivity contribute to variability in appetite and emotional regulation across sexes. Dumais and Veenema [69] describe region- and sex-specific patterns of OTR and vasopressin receptor (VPR) expression, implicating divergent neural mechanisms in males and females. Hormonal factors such as estrogen amplify OT’s behavioral effects, including its anxiolytic and potentially anorexigenic actions [25]. Since stress and anxiety modulate food intake, these interactions are highly relevant to appetite regulation. Vagal afferents also display neurochemical diversity, which may underlie sex-dependent functional differences. Powley et al. [129] highlighted distinct phenotypes within vagal neurons that could be selectively targeted. Given the localization of OTRs in vagal afferents and central regions, sex-based modulation of these circuits is plausible. Peripheral OT activates vagal afferents and suppresses food intake without requiring central penetration [36], supporting a peripheral site of action for sex-related differences. While current data show no dramatic sex divergence in VOR-mediated responses, subtle distinctions may be masked by study design. Future research should incorporate sex as a biological variable to better understand personalized applications of OT-based therapies in metabolic and GI disorders. To illustrate the diverse physiological effects mediated by vagal oxytocin receptors, Table 1 categorizes their known actions into four functional domains, along with underlying mechanisms and key experimental findings.

## 8. The Gut–Brain–Vagus Axis: A Systems Biology Perspective

### 8.1. Integration of Gut, Brain, and Vagus Nerve

The gut–brain–vagus axis forms a dynamic, bidirectional network that coordinates neural, hormonal, and immune pathways involved in digestive regulation and metabolic control [70]. It relies on vagal afferents and efferents to convey sensory and motor information between the gastrointestinal tract and central autonomic centers, influencing digestion, motility, and satiety [43,51]. Sensory fibers project into the nucleus tractus solitarius (NTS), where signals from gut distension, nutrients, and peptides such as cholecystokinin (CCK) are processed to influence hypothalamic centers and regulate appetite and energy expenditure [52]. Oxytocin modulates this axis by acting on vagal oxytocin receptors (VORs) located in nodose ganglia and the enteric nervous system, thereby influencing autonomic output, visceral sensitivity, and GI motor activity. Projections from the PVN to the brainstem further refine this regulatory loop [20,55]. While OT tends to reduce upper GI motility, it enhances colonic contractility in various species [20,71]. It also buffers stress-induced disturbances in GI function by modulating neuroendocrine circuits [48]. Dysregulation of these integrated pathways is linked to disorders such as irritable bowel syndrome, which blends altered motility with pain and mood disturbances. Additional layers of control involve sex hormones and microbial signals [129]. While VORs are promising therapeutic targets in dysphagia, obesity, and metabolic disease, further molecular and functional research is needed to fully characterize their roles across species [51,55,125].

Idiopathic gastroparesis (IGP), a functional GI disorder characterized by delayed gastric emptying without mechanical obstruction, shares several clinical and pathophysiological features with conditions such as irritable bowel syndrome and functional dyspepsia. Recent findings suggest that IGP may represent a subclinical form of vagal neuropathy or impaired central autonomic regulation [70]. Given that VORs influence both vagal afferent signaling and efferent control of gastric motility, their dysfunction could impair gastric reflexes and contribute to symptoms like nausea, bloating, and postprandial fullness. These observations support the idea that targeting peripheral VORs could be a promising strategy in managing gastroparesis and other vagally mediated dysmotility syndromes [70].

### 8.2. Influence of Microbiota on Vagal Oxytocin Signaling

The gut microbiota shapes vagal function by producing metabolites and immune signals that influence central processing. Compounds such as short-chain fatty acids (SCFAs) and pathogen-associated molecular patterns (PAMPs) modulate vagal afferents and brainstem circuits [10]. Oxytocin signaling is increasingly recognized as a mediator of microbiota–brain communication. In rodents, OT administration during stress not only reduces anxiety-like behavior but also reshapes microbiota composition, indicating a bidirectional feedback loop involving OT signaling and microbial ecology [4,56]. Microbiota-driven metabolites and immune cues may modify vagal sensitivity and receptor expression profiles, including OTR, potentially reprogramming neuronal responses. This reprogramming could affect vagal responses to GLP-1 and other gut-derived signals, though the specific contribution of OTR remains underexplored [127]. Microbiota-derived cues may also impact neuronal protein expression such as α-synuclein, hinting at broader implications for vagus-mediated brain function [10]. Due to the diversity of vagal subtypes and microbial inputs, high-resolution techniques like single-cell transcriptomics are essential to uncover how oxytocin and microbiota co-regulate gut–brain communication.

The composition and function of the gut microbiota are profoundly shaped not only by host genetics and disease states but also by extrinsic environmental factors. Diet, pollutants, antibiotic exposure, stress, and lifestyle choices such as physical activity and sleep patterns are increasingly recognized as key modulators of microbial diversity and gut barrier integrity [130]. These elements may indirectly influence vagal signaling and oxytocinergic regulation by altering microbial-derived metabolites and immune cues.

The microbiota’s influence on vagal oxytocin signaling operates through multiple interconnected pathways that extend beyond simple metabolite production. Specific bacterial strains produce neuroactive compounds that can directly modulate vagal tone and oxytocin receptor sensitivity in enteric neurons [19,116]. The gut microbiota also influences the expression and function of other neuropeptide systems that interact with oxytocin signaling, including ghrelin, GLP-1, and cholecystokinin pathways [8,19,115]. This creates a complex regulatory network where microbial changes can cascade through multiple neuroendocrine systems to alter gut–brain communication. Furthermore, the microbiota plays a crucial role in maintaining gut barrier integrity and immune homeostasis, both of which are essential for proper vagal oxytocin function [32,86,87]. Disruptions in microbial ecology, such as those occurring during dysbiosis or inflammatory conditions, can compromise the gut barrier and alter the local inflammatory environment, thereby modifying how vagal terminals respond to oxytocin and other signaling molecules [30,87,118].

The therapeutic implications of microbiota–vagal–oxytocin interactions are particularly relevant for personalized medicine approaches in gastrointestinal disorders. Individual variations in microbiome composition may explain some of the heterogeneity in oxytocin treatment responses observed in clinical populations [15,19]. Targeting the microbiota through probiotics, prebiotics, or fecal microbiota transplantation could potentially enhance the efficacy of oxytocin-based therapies by optimizing the microbial environment for vagal signaling [116]. Conversely, oxytocin treatments may themselves serve as microbiome-modulating interventions, creating beneficial feedback loops that support both gut health and neurological function [19]. The timing of microbiota-targeted interventions may be particularly important during critical developmental windows when both the microbiome and vagal oxytocin systems are being established, suggesting that early-life interventions could have lasting effects on gut–brain communication patterns [37,111]. Understanding these complex interactions will be essential for developing comprehensive treatment strategies that address the full spectrum of factors influencing vagal oxytocin signaling in health and disease.

### 8.3. Inflammation and Vagal Function

Inflammation profoundly impacts vagal circuits within the gut–brain axis, altering both gastrointestinal and emotional regulation. Proinflammatory mediators increase visceral afferent excitability and disrupt central processing, contributing to symptoms such as pain, nausea, and altered motility, as well as mood disturbances [131]. Inflammatory signals may impair vagal reflex control by altering afferent terminal activity, as seen in reflux and dysphagia [129,132]. Oxytocin signaling is sensitive to immunological and microbial changes, and has been shown to mitigate inflammation-induced behavioral and microbial disturbances [4]. Inflammatory disruption of brainstem–hypothalamic–cortical circuits, particularly in the amygdala and prefrontal cortex, may further affect OT-dependent regulation [65]. At the peripheral level, vagal efferents regulate motility via the myenteric plexus, a system vulnerable to inflammatory stress, especially within the dorsal motor nucleus of the vagus [34]. These findings highlight the relevance of inflammation–OT–vagal crosstalk in both metabolic and neuropsychiatric conditions [4,131].

### 8.4. Metabolic Stress and Plasticity of Vagal Circuits

Metabolic challenges such as obesity or caloric restriction induce plastic changes in vagal circuits that shape food intake, energy expenditure, and GI function [43,52]. OT helps mediate adaptive responses to these changes. In rodent models, peripheral OT administration reduces food intake and body weight, partly by activating vagal afferents that project into the hypothalamus. These effects are associated with increased c-Fos expression in PVN neurons, which is abolished after vagotomy, confirming the role of vagal OT pathways [36]. Stress-induced shifts in microbiota may feedback onto oxytocin-regulated vagal circuits, as shown by OT-driven changes in microbial populations (e.g., *Mogibacterium*) [4]. Hormonal signals such as leptin, insulin, and GLP-1 also act via both humoral and vagal routes to influence hypothalamic control centers [52]. Early-life inflammation may program vagal function through CRF-mediated mechanisms, potentially interacting with OT signaling [5]. OT has also been implicated in stimulating brown adipose tissue (BAT) thermogenesis and modulating enteric motility via OT-immunoreactive fibers in the gut [51,71,133,134]. Together, these findings illustrate how OT integrates microbial, endocrine, and environmental signals to reshape vagal plasticity and maintain metabolic homeostasis. These insights strengthen the therapeutic potential of targeting VORs in metabolic and gastrointestinal disorders [4,36,67].

## 9. Translational and Therapeutic Implications

### 9.1. Targeting Vagal Oxytocin Receptors in Gastrointestinal and Metabolic Disorders

Vagal oxytocin receptors have emerged as promising therapeutic targets for gastrointestinal and metabolic diseases due to their critical role in regulating motility, satiety, and thermogenesis [53,54]. These receptors are found on specialized vagal afferents, including intraganglionic laminar endings (IGLEs), mucosal terminals, and neurons in the nodose ganglia, forming the anatomical basis for oxytocin’s modulatory actions throughout the gut–brain axis. In disorders such as dysphagia or esophageal dysfunction, selectively targeting vagal afferents, rather than focusing solely on smooth muscle tone, may yield more effective results [129]. Similarly, in metabolic conditions, both central and peripheral oxytocin administration has demonstrated beneficial effects, including appetite suppression and weight loss, even in leptin-resistant or high-fat diet models [22,67]. Human studies report rapid appetite suppression with intranasal oxytocin, particularly in individuals with obesity [25]. Mechanistically, VORs mediate these effects by integrating hormonal signals (e.g., GLP-1), enhancing brown adipose tissue (BAT) thermogenesis, and influencing substrate utilization [51,88]. Importantly, oxytocin does not induce aversive reactions such as nausea or interfere with other behavioral domains, reinforcing its clinical appeal [62,135,136]. Still, challenges persist, including oxytocin’s short half-life, limited receptor specificity, and variability in vagal signaling across individuals [3,4]. Addressing these issues will require advanced tools such as single-cell transcriptomics, functional circuit mapping, and receptor-specific delivery technologies [68].

### 9.2. Drug Delivery and Ligand Design: Challenges and Opportunities

Effective therapeutic targeting of VORs is complicated by the anatomical diversity of the vagus nerve and the high structural similarity between oxytocin and vasopressin receptors, increasing the risk of off-target effects, particularly in sensitive brain regions like the amygdala [14,137]. The broad peripheral distribution of OTRs (e.g., the colon, hypothalamus, uterus, etc.) necessitates delivery systems that minimize systemic exposure while maximizing receptor specificity [24,46]. While tools like DREADDs and optogenetics offer precision in preclinical studies, their clinical use remains limited [7,138]. Poor blood–brain barrier (BBB) permeability of many oxytocin analogs further complicates delivery, prompting the use of intranasal routes, which have shown metabolic benefits in humans [72]. However, chronic use may induce receptor desensitization [63]. Innovative delivery platforms, including nanoparticles, slow-release systems, and viral vectors, are being explored to optimize specificity and pharmacokinetics [6,88]. Context-dependent receptor responses also complicate therapy: oxytocin can yield different outcomes depending on tissue type and hormonal milieu, particularly within the vagal system where it interacts with GLP-1 and CCK [34,133]. As metabolic responses vary by phenotype, individualized dosing strategies may be required. For instance, diet-induced obese models are more responsive to oxytocin, suggesting therapeutic efficacy could be optimized by tailoring dose to metabolic status [67,139,140,141,142,143,144,145,146]. Systems biology approaches, integrating transcriptomics, imaging, and computational modeling, will be crucial in refining ligand design and predicting treatment outcomes [27,62].

### 9.3. Peripheral Versus Central Targeting Strategies

**Figure 5:**
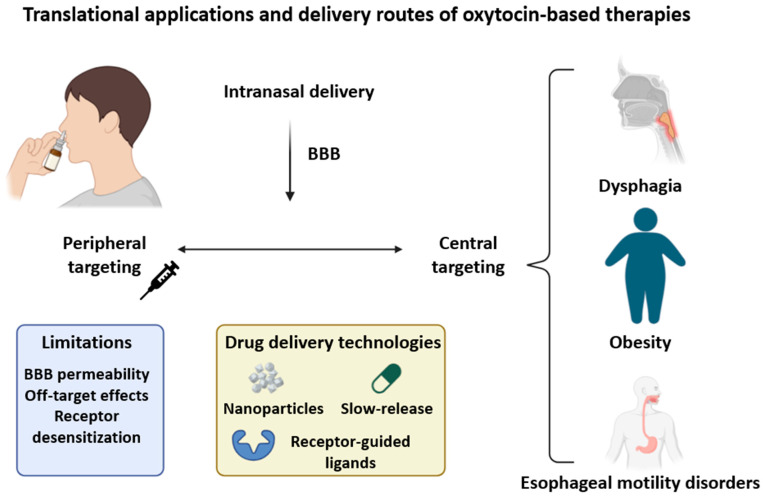
Translational applications and delivery routes of oxytocin-based therapies. Schematic illustration of current and emerging strategies for delivering oxytocin to modulate vagal oxytocin receptors. Intranasal administration allows partial blood–brain barrier (BBB) penetration, offering access to central circuits. Peripheral approaches, including systemic injections, affect vagal afferents and enteric sites. Therapeutic applications include dysphagia, obesity, and esophageal motility disorders. Limitations include low BBB permeability, off-target effects, and receptor desensitization. Nanoparticles, slow-release formulations, and receptor-guided ligands represent promising delivery technologies to enhance efficacy and specificity.

Peripheral and central approaches offer distinct advantages for modulating VORs. Peripheral delivery methods (e.g., intranasal or systemic administration) are non-invasive and can influence CNS circuits via indirect routes such as blood transport or vagal afferents [63,72]. Intranasal oxytocin reaches hypothalamic regions and produces behavioral effects, although the exact mechanism and sex-specific differences remain uncertain [147]. More direct engagement of CNS pathways, such as via intracerebroventricular injection, allows targeted modulation of brainstem circuits like the dorsal motor nucleus of the vagus (DMNV), where oxytocin enhances neuronal excitability [56]. Optogenetic evidence further supports the functional role of VORs in central autonomic regulation. OTRs are also present in enteric structures, including the mucosa and myenteric plexus, suggesting that combined delivery routes may offer synergistic benefits [5,7,36,148,149]. Peripheral administration, especially intranasal, has demonstrated efficacy in reducing appetite and improving metabolic markers, though its effects may be attenuated by enzymatic degradation or limited BBB penetration. Central delivery methods may be preferable in refractory cases, allowing more precise circuit engagement. Importantly, peripheral oxytocin can still influence central pathways via vagal or sympathetic routes, as shown by its ability to enhance thermogenesis and modulate adrenergic signaling [69,132,149,150,151,152,153]. Given the widespread distribution and context-dependent responses of VORs, integrating peripheral and central strategies may maximize therapeutic impact. Advancements in ligand targeting, nanocarriers, and tissue-specific delivery are likely to enhance clinical translation [5,56]. To illustrate how oxytocin-based therapies can be applied in clinical settings, Figure 5 summarizes current delivery routes, associated challenges, and their relevance to treating vagus-related disorders such as dysphagia, obesity, and esophageal motility disturbances.

## 10. Future Directions and Open Questions

### 10.1. Technological Innovations for Vagal Oxytocin Research

#### 10.1.1. Spatial Transcriptomics and Single-Nucleus RNA Sequencing

Advances in spatial transcriptomics and single-nucleus RNA sequencing (snRNA-seq) have revolutionized our ability to study oxytocin signaling within the vagal system. Unlike traditional methods such as immunohistochemistry, which are limited by cross-reactivity and tissue preservation issues [68], these transcriptomic techniques allow high-resolution mapping of oxytocin receptor (OTR) expression in anatomically defined regions like the dorsal vagal complex (DVC) and dorsal motor nucleus of the vagus (DMV). Spatial transcriptomics preserves tissue architecture, enabling the detection of OTR mRNA alongside neurotransmitters such as GABA or glutamate [97]. Complementarily, snRNA-seq profiles individual nuclei, even from frozen tissues, making it suitable for rare cell types. Together, these tools help define the molecular identity of vagal oxytocin receptor (VOR)-expressing neurons and their potential roles in gut–brain communication. Functional markers like c-Fos, in response to oxytocin exposure, further bridge gene expression with activity [154]. Despite their utility, challenges persist, including the need for more specific antibodies and reliable OTR ligands for validation [4]. Integrating transcriptomic, genomic, and proteomic data may also reveal how genetic variability affects oxytocin sensitivity [155]. Ultimately, these approaches may uncover novel therapeutic targets in conditions such as obesity and dysphagia [22,24,133].

#### 10.1.2. Optogenetic and Chemogenetic Approaches

Optogenetics and chemogenetics have opened new avenues for studying VOR function with cell-type specificity. Light-based activation of oxytocinergic projections from the paraventricular nucleus (PVN) to the DMV enables real-time control over gut–brain circuits. In chronic stress models, such as intermittent hypoxia and hypercapnia (CIH/H), optogenetic stimulation of PVN neurons restores oxytocin release and improves vagal tone [14]. Chemogenetics, using DREADDs, offers an alternative with sustained activation. These tools have been instrumental in identifying GLP1R-expressing vagal neurons and their integration with brainstem centers [62]. Dual opto-chemogenetic studies have shown that oxytocin release enhances cardiac vagal tone and reverses CIH/H-related dysregulation via PVN–DMV–CVN circuits. While these models offer precise circuit dissection, they may not fully mimic endogenous signaling. Future research should integrate these tools with functional imaging and molecular profiling to refine our understanding of VOR-mediated pathways [88].

#### 10.1.3. Advanced Imaging and Functional Mapping

High-resolution imaging and mapping techniques are essential for characterizing VOR connectivity and function. Viral vectors expressing fluorescent proteins like tdTomato, combined with oxytocin immunolabeling, have enabled visualization of brainstem oxytocin neurons and their synaptic partners [43]. Electrophysiological recordings in DMV neurons, paired with optogenetic stimulation, have shown how subtle inputs modulate pacemaker activity [34]. Genetically encoded calcium indicators offer further insight into how VOR-expressing neurons respond to appetite-related stimuli. These tools help link neural activity with behavior and gut function. Tracing studies have confirmed PVN–DVC connectivity and highlighted roles for corticotropin-releasing factor (CRF) in stress responses [65]. Evidence also points to retrograde signaling in PVN–DMV communication [14]. Despite these advances, translation to humans remains limited by the lack of selective ligands and reliable in vivo imaging tools [4].

### 10.2. Key Unanswered Questions

Although mapping of VOR expression has progressed, several key questions remain. The identity and function of VOR-expressing vagal subtypes are unclear, including their relation to GLP1R-positive neurons and whether they have distinct sensory modalities [88,106]. How VOR activation influences appetite regulation and esophageal motor function is also not fully understood. The involvement of neurons in the nucleus tractus solitarius (NTS), including those expressing POMC or GLP-1, warrants investigation. While oxytocin plays a role in gut–brain signaling, the contribution of VORs specifically, and their independence from other peptides, remains uncertain. Translational challenges include variability across species, the limited availability of human data, and underexplored sex differences [3]. Current tools such as Cre/LoxP systems and calcium imaging have helped, but more refined techniques are needed to isolate and study VORs in human tissue [106].

### 10.3. Translational Potential and Pathways to Clinical Application

Harnessing VOR signaling for therapeutic use requires a comprehensive understanding of its physiological roles and pharmacology. As noted in previous sections, oxytocin affects appetite, motility, and energy balance [36,67,156]. However, determining the relative contributions of central and peripheral pathways is essential for designing targeted interventions. Intranasal delivery has shown promise in preclinical obesity models [128], but species-specific receptor differences and inconsistent outcomes highlight the need for improved human-relevant models [68]. VOR-targeted therapies may benefit conditions like dysphagia and functional GI disorders, especially where standard treatments fail [34,46]. Addressing sex differences in oxytocin signaling will be critical for precision medicine approaches [23]. Systems biology tools, including omics and computational modeling, could aid in identifying biomarkers and developing selective ligands.

### 10.4. Mechanistic and Translational Gaps in VOR-Mediated Appetite Control

Despite encouraging findings, key mechanistic gaps persist in our understanding of VORs in appetite regulation. Although expression has been confirmed in vagal afferents and enteric neurons, species variability in the DVC and DMV complicates interpretation [14,54]. Integration with other gut peptides like GLP-1 and nesfatin-1 is poorly characterized [36]. Peripheral contributions to appetite suppression, suggested by vagotomy studies, vary with dose, sex, and study design [3]. Chronic oxytocin exposure may lead to receptor desensitization, yet its molecular basis remains unclear. Translating findings to humans is further hampered by limited anatomical data and challenges in modeling sex and psychosocial variables. Moving forward, combining transcriptomics, imaging, and patient-derived models will be crucial to understand VOR biology and develop effective therapies for obesity, dysphagia, and GI disorders.

## 11. Summary of Key Studies on Vagal Oxytocin Signaling

To enhance clarity and aid interpretation, we provide a summary of representative studies discussed in this review (Table 2). The table highlights the experimental models used (both preclinical and clinical), key focus areas, and principal findings related to vagal oxytocin signaling along the gut–brain axis.

## 12. Conclusions

The vagal oxytocin receptor (VOR) system has emerged as a key integrator of gut–brain communication, linking sensory input from the gastrointestinal tract with central autonomic, endocrine, and behavioral control. Evidence from molecular, anatomical, and functional studies supports its involvement in modulating esophageal motility, gastrointestinal function, satiety signaling, and energy balance. VORs are strategically located in vagal afferents, brainstem nuclei, and the enteric nervous system, where they interact with a broad array of neuromodulators and gut-derived peptides. Despite significant advances, critical questions remain regarding the cell-type specificity, spatial distribution, and plasticity of VOR-expressing neurons, particularly in humans. The integration of oxytocin signaling with other metabolic pathways, such as GLP-1, insulin, and gut microbiota-derived signals, adds further complexity to this regulatory network. In addition, sex-based variability and translational gaps between animal models and human physiology must be addressed to inform effective therapeutic strategies. Technological innovations, including spatial transcriptomics, optogenetics, and advanced circuit mapping, offer unprecedented resolution for dissecting the structure and function of VOR pathways. These tools are poised to drive discovery of new therapeutic targets for conditions such as dysphagia, obesity, and functional gastrointestinal disorders. Yet, challenges related to receptor selectivity, pharmacokinetics, and delivery methods, especially across the blood–brain barrier, must be overcome to enable clinical translation. Altogether, VORs represent a promising but underexplored target within the gut–brain axis. Continued interdisciplinary research is essential to resolve remaining mechanistic gaps and realize the therapeutic potential of modulating this multifaceted system in gastrointestinal and metabolic diseases.

## Figures and Tables

**Table 1:** Functional roles of vagal oxytocin receptors in appetite control and metabolic regulation.

Domain	Mechanisms and Effects	Key Findings
Satiety signaling	– Peripheral OT acts via VORs in nodose ganglia and NTS – Enhances vagal afferent input to the brain – Integrates mechanical (stretch) and chemical cues from the GI tract	– OT reduces food intake in animals and humans – VORs are essential for satiety and vagal signaling
Meal patterns	– VORs regulate meal size, frequency, and timing – Respond to GLP-1, nesfatin-1, and gut stretch – May influence macronutrient selection	– OT reduces meal size and frequency – Disrupted vagal signaling affects meal organization
Metabolic regulation	– Promotes thermogenesis and fat oxidation – Improves glucose and lipid metabolism – Acts synergistically with nesfatin-1 and GLP-1	– OT improves metabolic markers – VORs link gut signals to energy expenditure
Sex differences	– VOR distribution and signaling may vary by sex – Estrogen enhances OT action – Vagal neuron phenotypes may underlie sex-specific responses	– Sex-dependent differences reported – Further studies should include sex as a biological variable

Abbreviations: OT—oxytocin; VOR—vagal oxytocin receptor; NTS—nucleus tractus solitarius; GLP-1—glucagon-like peptide-1; GI—gastrointestinal.

**Table 2:** Summary of key studies on vagal oxytocin signaling and gut–brain axis regulation.

Study	Model	Study Type	Key Characteristics	Main Findings	Quality/Limitations
[3]	Rat	Experimental	Methamphetamine self-administration; peripheral OT administration	Vagus nerve mediates OT’s suppressing effects on drug seeking behavior	High quality; specific behavioral paradigm
[5]	Mouse/Rat	Histological/Developmental	Expression of OT/OTR in enteric nervous system across development	OT and OTR widely expressed in ENS and intestinal epithelium	High quality; comprehensive developmental analysis
[6]	Mouse	Experimental/Genetic	Vagal OTR mouse model; esophageal motility assessment	Vagal OTRs necessary for normal esophageal motility and function	High quality; genetic approach with functional outcomes
[7]	Rat	Electrophysiological	Stress-induced gastric motility; hypothalamic–vagal circuitry	OT modulates gastric emptying via hypothalamic–vagal pathways following stress	High quality; mechanistic approach
[14]	Rat	Physiological	Chronic intermittent hypoxia model; cardiovascular outcomes	OT neuron activation prevents hypertension in sleep apnea model	Good quality; clinically relevant model
[20]	Rat	Pharmacological	Distal colon motility; nitrergic mechanisms	OT inhibits colonic contractions via NO-cGMP-K+ channel pathway	High quality; detailed mechanistic analysis
[21]	Mouse	Pharmacological/Behavioral	Central 5-HT mediation; colonic motility assessment	Central 5-HT mediates colonic motility through hypothalamic OT–colonic OTR pathway	Good quality; dual central–peripheral approach
[22]	Rat	Metabolic	Diet-induced obesity model; peripheral OT administration	Peripheral OT suppresses food intake and causes weight loss in obese rats	High quality; clinically relevant obesity model
[23]	Human	Clinical trial	Single-dose intranasal OT in healthy men	OT reduces caloric intake in men	Moderate quality; small sample, single-dose design
[24]	Rat	Neurophysiological	NTS OTR signaling; feeding behavior	Endogenous OTR signaling in NTS controls satiation and thermogenesis	High quality; specific brain region focus
[25]	Human	Clinical trial	Post-stress eating in women; intranasal OT	OT reduces sweet snack intake without affecting cortisol	Good quality; gender-specific, stress paradigm
[27]	Mouse	Metabolic	Obese mouse model; thermogenesis assessment	OT improves metabolic dysfunction via increased thermogenesis	Good quality; mechanistic metabolic focus
[30]	Mouse	Pharmacological	GI motility, inflammation, permeability assessment	OT regulates multiple GI functions including motility and inflammation	High quality; comprehensive GI analysis
[36]	Mouse	Neuroanatomical	Peripheral-to-central OT relay via vagal afferents	Peripheral OT activates central OT neurons via vagal pathway for feeding control	High quality; novel relay mechanism identified
[53]	Mouse	Pharmacological	Vagal afferent activation; feeding behavior in normal and leptin-resistant mice	Peripheral OT activates vagal afferents to suppress feeding in both normal and leptin-resistant states	High quality; clinically relevant leptin resistance model
[59]	Rat	Electrophysiological	Vagal afferent fiber role in OT-induced gastric modulation	Vagal afferents determine OT-induced gastric tone modulation	High quality; direct neural recording approach
[60]	Rat	Pharmacological	NTS OTR signaling; satiation signal processing	NTS OTR signaling processes GI satiation signals for food intake control	High quality; specific satiation mechanism focus
[48]	Rat	Stress model	Water-avoidance stress; colonic motility	OT inhibits stress-induced accelerated colonic motility	Good quality; stress-specific GI effects
[49]	Rat	Pharmacological	Gastric smooth muscle; motility assessment	OTRs on gastric smooth muscle mediate excitatory effects on motility	Good quality; direct tissue-level analysis
[54]	Mouse	Genetic/Optogenetic	Vagal sensory neuron identification and manipulation	Genetic identification of specific vagal neurons controlling feeding	High quality; cutting-edge genetic tools
[58]	Rat	Physiological	Forestomach pressure; dorsal vagal complex involvement	OT increases intragastric pressure via dorsal vagal complex	Good quality; specific gastric region focus
[71]	Human	Clinical	Healthy women; colonic motor activity assessment	OT stimulates colonic motor activity in healthy women	Good quality; gender-specific clinical data
[72]	Human	Clinical trial	Obese vs. normal-weight men; food intake assessment	OT’s anorexic effects stronger in obese than normal-weight men	Good quality; BMI-stratified analysis
[128]	Prairie vole	Metabolic	Diet-induced obesity model; intranasal OT treatment	Intranasal OT reduces weight gain in diet-induced obese prairie voles	Moderate quality; non-traditional rodent model
[133]	Rat	Pharmacological	Chronic hindbrain OT administration; weight loss	Chronic hindbrain OT sufficient to elicit weight loss in obese rats	High quality; chronic treatment paradigm
[154]	Mouse	Metabolic	Peripheral OT treatment; obesity and food intake	Peripheral OT ameliorates obesity by reducing food intake and visceral fat	Good quality; comprehensive metabolic assessment
[144]	Rat	Pharmacological	Combined OT and naltrexone treatment; feeding behavior	Subthreshold OT–naltrexone combination affects feeding and brain gene expression	Good quality; novel combination therapy approach

## Data Availability

No new data were created or analyzed in this study. Data sharing is not applicable to this article.
